# Intrafractional dose variation and beam configuration in carbon ion radiotherapy for esophageal cancer

**DOI:** 10.1186/s13014-016-0727-2

**Published:** 2016-11-15

**Authors:** M. F. Haefner, F. Sterzing, D. Krug, S. A. Koerber, O. Jaekel, J. Debus, M. M. Haertig

**Affiliations:** 1 Department of Radiation Oncology, Heidelberg University Hospital, Im Neuenheimer Feld 400, 69120 Heidelberg, Germany; 2 National Center for Radiation Research in Oncology (NCRO), Heidelberg Institute for Radiation Oncology (HIRO), Im Neuenheimer Feld 400, 69120 Heidelberg, Germany; 3 Department of Radiation Oncology Kempten, Robert-Weixler-Straße 50, 87439 Kempten, Germany; 4 Department of Medical Physics in Radiation Oncology, German Cancer Research Center (dkfz), Im Neuenheimer Feld 280, 69120 Heidelberg, Germany; 5 Department of Radiation Oncology Baden-Baden, Balger Straße 50, 76532 Baden-Baden, Germany

**Keywords:** Esophageal cancer, Particle radiotherapy, Carbon ion radiotherapy, Organ motion, Dose robustness

## Abstract

**Background:**

In carbon ion radiotherapy (CIR) for esophageal cancer, organ and target motion is a major challenge for treatment planning due to potential range deviations. This study intends to analyze the impact of intrafractional variations on dosimetric parameters and to identify favourable settings for robust treatment plans.

**Methods:**

We contoured esophageal boost volumes in different organ localizations for four patients and calculated CIR-plans with 13 different beam geometries on a free-breathing CT. Forward calculation of these plans was performed on 4D-CT datasets representing seven different phases of the breathing cycle. Plan quality was assessed for each patient and beam configuration.

**Results:**

Target volume coverage was adequate for all settings in the baseline CIR-plans (V_95_ > 98% for two-beam geometries, > 94% for one-beam geometries), but reduced on 4D-CT plans (V_95_ range 50–95%). Sparing of the organs at risk (OAR) was adequate, but range deviations during the breathing cycle partly caused critical, maximum doses to spinal cord up to 3.5x higher than expected. There was at least one beam configuration for each patient with appropriate plan quality.

**Conclusions:**

Despite intrafractional motion, CIR for esophageal cancer is possible with robust treatment plans when an individually optimized beam setup is selected depending on tumor size and localization.

## Background

Radiotherapy is a central component of neoadjuvant or definitive concepts in multimodality treatment of esophageal cancer [1, 2]. Despite several different approaches to improve outcome over the past years by treatment intensification through radiation dose escalation or the addition of novel systemic agents [3, 4], a major improvement has not been accomplished yet resulting in a constantly high mortality rate for esophageal cancer patients.

For standard photon-based irradiation, intensity-modulated radiotherapy (IMRT) is recommended to provide a reasonable dose conformity to the target volume [5] and has facilitated integrated boost concepts in definitive treatment regimens [6]. Charged particle radiotherapy with carbon ions has been introduced as a new approach to improve radiooncological treatment strategies with a high relative biological effectiveness (RBE) and a high linear energy transfer (LET) compared to conventional photon-based irradiation. Clinical benefit of carbon ion radiotherapy (CIR) has already been demonstrated for other tumor entities [7, 8]. For esophageal cancer, there are some in vitro studies with CIR [9–11] as well as one clinical phase I/II trial from Japan showing first encouraging results [12]. Studies with proton radiotherapy for lung tumors revealed a big impact of organ and tumor motion on intrafractional dose distribution in particle irradiation potentially resulting in a severe underdosage of the target volume [13, 14].

The purpose of this study is to generate a better understanding of the effects of organ and target motion in carbon ion radiotherapy for esophageal cancer and to identify appropriate treatment planning settings for different target localizations within the esophagus providing adequate dose robustness, target volume coverage and sparing of the organs at risk (OAR). Our study uses CIR as a boost treatment of the primary tumor on the basis of beneficial bimodal treatment in other entities [8] and preparative to a clinical trial combining a carbon ion boost and elective IMRT of the lymphatic pathways.

## Methods

Preceding data collection, the study was approved by the institutional ethical review committee.

### Patients and atasets

We retrospectively selected four 4D-CT datasets from patients that have been treated with stereotactic body radiotherapy (SBRT) for lung malignancies at our institution (patient 1: 81 year old female, lung cancer in upper right lobe with 19 mm maximum diameter; patient 2: 90 year old male, lung metastasis from renal cell carcinoma in lower left lobe with 65 mm maximum diameter; patient 3: 81 year old female, lung metastasis from rectal cancer in upper right lobe with 39 mm maximum diameter; patient 4: 71 year old male, lung cancer in lower right lobe with 17 mm maximum diameter). Each of these CT datasets consisted of one free-breathing planning CT and seven co-registered 4D-CTs in different phases of the breathing cycle (three inspiratory: In25%, In50%, In75%; four exspiratory: Ex0%, Ex40%, Ex70%, Ex100%). CT scans were performed with patients in supine position with elevated arms and without abdominal compression. Slice thickness was 3 or 5 mm for the planning CT and 3 mm for the 4D-CT scans. Comparability of Hounsfield Units (HU) between planning CT and 4D-phases was guaranteed by calculating histograms of HU values to ensure a minimal systematic range deviation error.

### Target volumes and constraints

For each patient a certain section of the esophagus was defined as fictive gross tumor volume (GTV) on the free-breathing planning CT representing esophageal cancer. Consecutively, we added a caudal and cranial margin of 20 mm and a circumferential margin of 10–15 mm to obtain a boost planning target volume (PTV). To simulate variable planning scenarios with altering topographic conditions, target volumes were defined in different sizes and locations (patient 1: upper esophagus/cranio-caudal GTV extent: 20 mm/PTV volume: 47.8 ml; patient 2: middle esophagus/cranio-caudal GTV extent: 50 mm/PTV volume: 92.3 ml; patient 3: lower esophagus/cranio-caudal GTV extent: 50 mm, PTV volume: 91.7 ml; patient 4: lower esophagus/cranio-caudal GTV extent: 70 mm/PTV volume: 223.3 ml). Organs at risk (OAR) such as lung, heart, stomach or spinal cord were contoured on the free-breathing planning CT. All mentioned structures were consecutively transferred to the different 4D-CTs by elastic registration with minor manual corrections afterwards. Contouring and transformation of structures was performed with a dedicated software (OnQ rts v2.0, Oncology Systems Limited, Shrewsbury, UK).

### Treatment planning

**Fig. 1:**
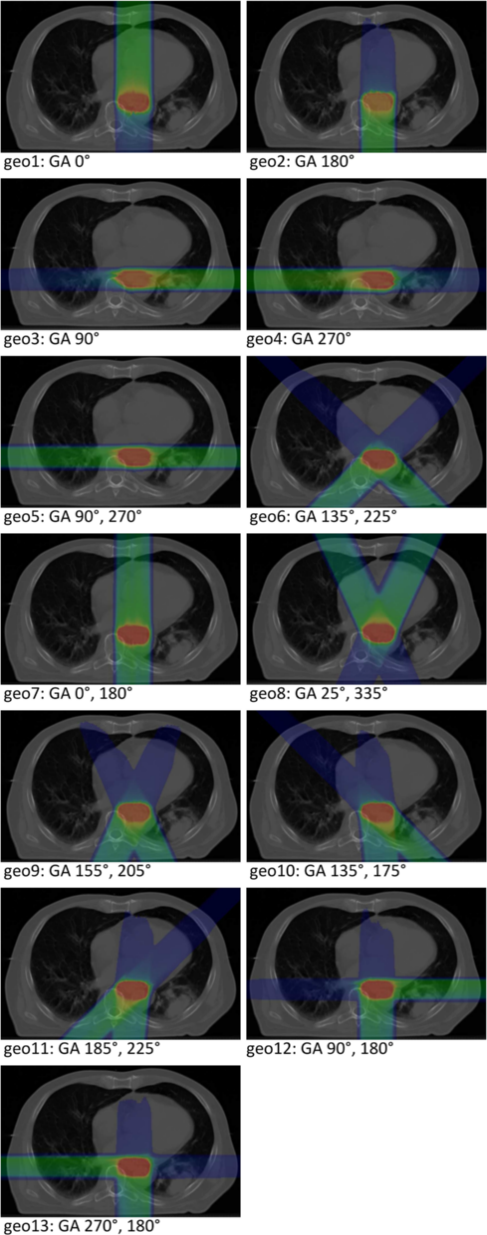
Overview of all 13 beam geometries with specifications of the according gantry angles (GA)

Treatment plans with 13 different field geometries (geometry 1–13; 4× one field, 9× two fields; couch fixed, gantry angles varied) were generated (Fig. 1) for each patient on the planning CT. Optimization of the fluences were performed with TriP-98 (GSI Helmoltzzentrum fuer Schwerionenforschung, Darmstadt, Germany) using the all-points dose calculation algorithm and low-dose LEM (local effect model) approximation. In two-field geometries, both fields were weighted equally. Secondly, forward dose calculation was performed separately for each 4D-CT (‘quasi-static’). Hence, no motion interplay was introduced. Planning CT and 4D-CT were acquired in the same imaging session, therefore the patient was in the same coordinate system. Correct position of the bony anatomy was reviewed. Forward calculation of the treatment plan was performed with TRiP-98 using the unmodified plan created in the optimization and using the same target point as before. Because of the large target volume of patient 4 we subsampled the dose calculation grid in the optimization step and then returned to full dose calculation in the forward calculation. Smearing margins technique was not used.

### Plan evaluation

Indices for PTV dosage were the volume that receives at least 95% of the prescribed dose (PTV V_95%_), the volume that receives more than 107% of the prescribed dose (PTV V_107%_), the median dose (PTV D_median_), the maximum dose (PTV D_max_), conformity index (CI) and homogenity index (HI). CI [15] and HI [16, 17] were defined as follows:

$\mathrm{C}\mathrm{I}=\frac{{\displaystyle \int dVD\kern0.5em \left(x,y,z\right)}}{{\displaystyle \int dVD\kern0.5em \left(x,y,z\right)}},0<CI<1$; (Integrated dose in PTV divided by integrated dose in whole body).

$\mathrm{H}\mathrm{I}=\frac{D_5-{D}_{95}}{D_{Prescribed}}$; (D_5%_ - D_95%_ in PTV divided by prescribed dose D_P_ , modified based on Wu et al. [16]).

Further plan quality criteria were defined by compliance with constraints for relevant OAR: the lung volume receiving 20% of the prescribed dose or less (V_20%_) < 20%, spinal cord D_max_ < 60%, mean heart dose < 30% for PTVs with major height overlap with the heart volume (i.e. tumors located in the distal esophagus) and < 10% for PTVs with minor or no height overlap (i.e. proximal tumors).

To carve out the optimal beam geometry for each patient the first step included the selection of six geometries with the best characteristics considering coverage/conformity and homogeneity. In a second step, these six geometries were evaluated by performance of sparing the OARs. In case of multiple geometries meeting all constraints importance ranking of the OARs was: lungs > heart > spinal cord. The optimal beam configuration for each patient was defined as the geometry in the top spot after this two-step evaluation process.

## Results

### Static planning CT

PTV V_95%_ was > 98% for all two-field geometries and > 94% for all one-field geometries. PTV V_107%_ was < 0.3% for all two-field geometries and < 1.2% for all one-field geometries. PTV D_median_ was 99.6 ± 0.5% of the prescribed dose independent from geometry. PTV D_max_ was < 120% of the prescribed dose except for Geometry 2 (122%) and Geometry 11 (133 %) in patient 4. CI was > 0.82 for all one-field geometries and >0.90 for all two-field geometries. HI was < 9.5% for all one-field geometries and < 6% for all two-beam geometries. In terms of OAR sparing, the lung V_20%_ was < 22% in all settings. Further, D_max_ of the spinal cord was < 78% of the prescribed dose for all plans. Mean heart dose was < 1% for patient 3 and < 37% for all other patients.

### 4D-CTs

**Fig. 2:**
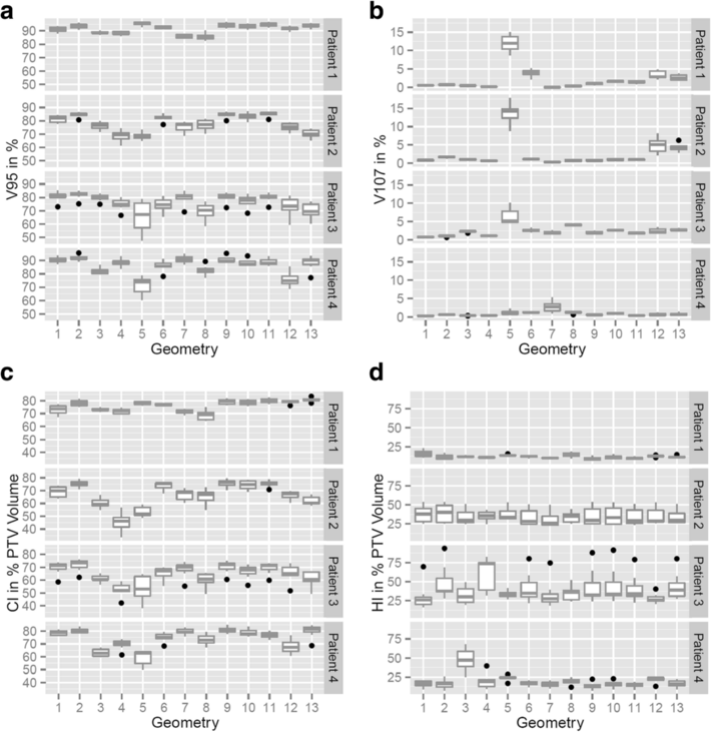
Boxplots for PTV-related **a**) V_95%_ **b**) V_107%_ **c**) conformity index (CI) and **d**) homogeneity index (HI) for every patient as a function of beam geometry including dosimetric information from treatment plans of all seven 4D-CT phases

PTV V_95%_ was reduced compared to the planning CT and ranged between 50 and 95 % in patients 2, 3 and 4 and 80–98% in patient 1. In particular, geometries 5, 8, 12 and 13 performed poorly. PTV V_107%_ was below 7% in most cases, except for geometry 5, which showed hot spots in up to 18% of the target volume. PTV Median dose was 98.3 ± 3.3% and mean dose was 96.7 ± 6.7%. PTV D_max_ ranged between 110 and 123.3 % for most cases but could reach up to 136.7% (patient 4, geo11). Conformity Index was reduced, patient 1: CI 65–85%, patients 2 and 3: CI 35–80%, patient 4: CI 50–85%. In geometries 1, 2, 9, 10 and 11, the CI is > 65% for all patients. Homogeneity Index is also depending on the patient, patient 1: 5–25%, patient 2: 25–50%, patient 3: 15–80%, patient 4: 5–25% (except for geometry 3 with up to 70%). Including all 4D-CT phases Fig. 2 shows boxplots for the mentioned PTV parameters according to beam geometry for all patients.

**Fig. 3:**
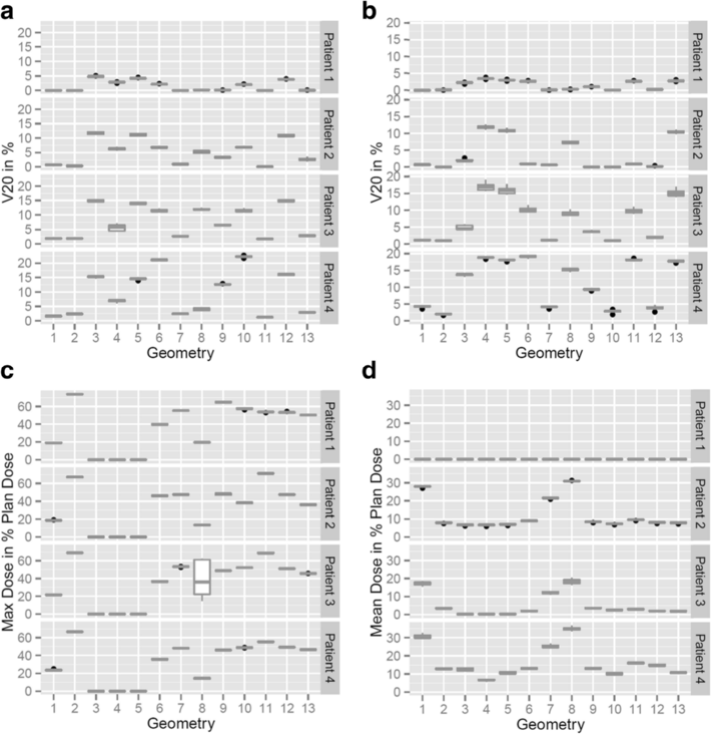
Boxplots for **a**) V_20%_ of the left lung **b**) V_20%_ of the right lung **c**) D_max_ of the spinal cord and **d**) mean heart dose for every patient as a function of beam geometry including dosimetric information from treatment plans of all seven 4D-CT phases

**Fig. 4:**
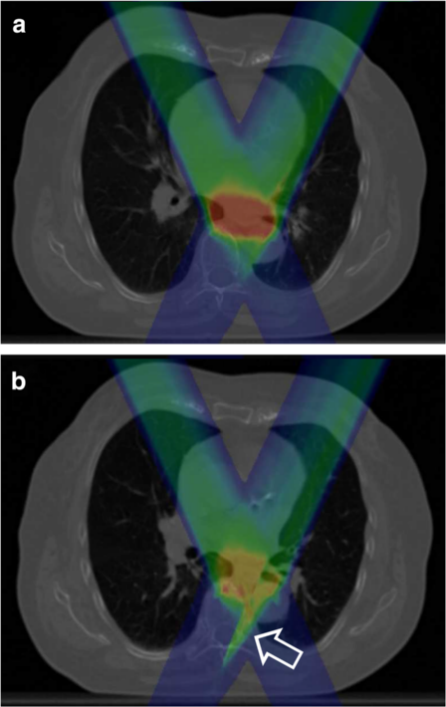
Dose distribution for patient 3 with geo8 calculated **a**) on the free-breathing planning CT and **b**) on the 4D-CT phase Ex0% with a critical overshoot due to slight variations of the left heart contour (*arrow*)

Boxplots for the relevant OAR constraints depending on beam geometry and breathing phase are shown in Fig. 3. For all geometries and 4D-CT phases the relevant OAR dose parameters did not diverge significantly from those calculated for the static planning CT except for D_max_ of the spinal cord in some scenarios. Figure 4 exemplifies a significant dose overshoot due to a minor heart deformation in the 4D-CT (patient 3, geo8, phase Ex0%) compared to the initial planning CT dose distribution resulting in a critical increase of D_max_ in the spinal cord from expected 16.7 % of the prescribed dose to up to 60%.

### Optimal beam configurations

**Fig. 5:**
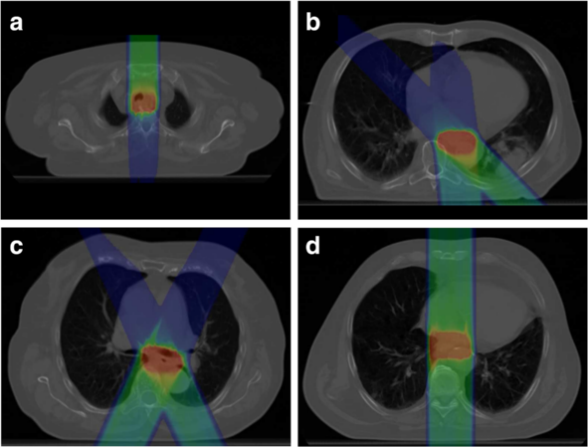
Optimal beam configuration for **a**) patient 1 (geo1), **b**) patient 2 (geo10), **c**) patient 3 (geo9) and **d**) patient 4 (geo7)

Geometries 1, 2, 7, 9, 10 and 11 were universally found to result in reliable treatment plans for all patients, based on PTV V_95%_ and Conformity Index, the largest difference being the dose to the organs at risk. Taking this into account, geometry 1 yields the optimal one-field treatment plan for each patient, with low doses to lungs and spinal cord and tolerable mean doses to the heart. Using two fields, the optimal plan configurations were more dependent on the individual patient and PTV location: geometry 10 for patient 2, geometry 9 for patient 3 and geometry 7 for patient 4 (Fig. 5). In patient 1, geometry 5 would be the optimal two-field treatment plan, but it is outperformed by the single-field treatment plan (geometry 1) due to a large PTV D_max_ and PTV V_107%_.

## Discussion

The free-breathing planning CT is rather a merge of different anatomical situations over the breathing cycle than specifically corresponding to one of the quasi-static 4D phases. Consequently, the general reduction of plan quality in all forward calculated 4D-plans regardless of the beam geometry was expected. However, the dimension of quality loss strongly varied depending on target volume localization and beam configuration and mainly affected PTV-related parameters such as coverage or adequate dosage. In comparison, the impact on sparing of the organs at risk was slightly lower, especially for mean doses of parallel OAR, though high maximum doses to the spinal cord could be a crucial problem.

We presented one favored beam geometry for each patient or tumor site, respectively. However, there were further, suitable beam sets with only minimal limitations compared to the optimal plan configuration. Accordingly, there is a selection of beam configurations for each tumor localization along the esophagus and specific anatomy and tumor characteristics may contribute to determining the optimal plan setting for each individual patient. Basically, strictly anterior or posterior beam directions are favorable for tumors localized in the upper two thirds of the esophagus whereas a combination of dorsal beam angles may be advantageous for targets in the distal esophagus when it comes to optimizing the mean heart dose.

Intrafractional motion-induced variations potentially result in geometric misses because of target structures leaving the high dose area. This problem concerns both photon-based and particle radiotherapy. From several studies based on 4D-CT measurements or fiducial-based approaches we know that especially the distal esophagus is mobile in the cranio-caudal direction up to 16 mm dependent on breathing motion [18–21]. In an analysis of gross tumor volume (GTV) and internal tumor volume (ITV) of carcinomas of the gastro-esophageal junction, Zhao et al. showed that ITVs were 72% larger on average and up to 172% larger at maximum than the corresponding GTVs and proposed asymmetrical margins up to 16 mm inferior towards the stomach [22].

Further, motion-induced variations are a particular difficulty for particle radiotherapy due to altering density profiles alongside the radiation beam track resulting in range deviations and displaced spread-out Bragg peaks (SOBP). Especially the thoracic topography with moving and close-by located tissues of low (lung) and rather high density (e.g. heart or diaphragm) puts up a challenge for creating robust radiation treatment plans. Severe range deviations may lead to critical dose peaks in OAR as demonstrated for patient 2 (Fig. 4). Similar effects have been shown by Zhang et al. for proton irradiation due to different stomach gas fillings with D_max_ increases of more than 10% to the spinal cord [23].

Several different approaches in particle radiation planning have been introduced to control the mentioned problems. The gating technique has predominantly been established for photon-based irradiation and might be a promising approach in CIR of moving tumors combined with rescanning [24, 25]. Other compensation strategies include dose smearing as analyzed for proton irradiation of lung tumors [14, 26] or increasing the beam spot size which has demonstrated a potential compensatory benefit in CIR of liver tumors [27]. Some study groups have also investigated dose calculations on different planning CT phases for proton radiotherapy of lung tumors and presented suitable solutions [13, 14, 26]. However, there is lack of data concerning CIR planning for esophageal tumors in particular. We were able to show that the cornerstone for a robust treatment plan for these patients is accurate target volume definition and appropriate beam configuration respecting the individual anatomy, both based on 4D-CT information.

Of course, in the evaluation of treatment quality interfractional variations have to be considered as well. Internal changes in the patient such as location variability of the target structures, tumor response with consecutive volume reduction or non-tumor-related variations (e.g. pleural effusion) may lead to critical range deviations. Additionally, daily image guidance is recommended for photon-based radiotherapy [28] and mandatory for particle irradiation.

Pulmonary toxicity is one of the major problems in esophageal irradiation and increases perioperative morbidity in the neoadjuvant setting dependening on the radiation dose applied to the lungs [29] In particular, the volume that receives doses of 5 Gy or less is an important risk factor for the development of pulmonary complications [30]. Compared to intensity-modulated radiotherapy (IMRT), particle radiotherapy has the potential benefit to significantly reduce the lung volume receiving lower doses. For a PTV dose of 50.4 Gy and a GTV dose escalation up to 65.8 Gy, Welsh et al. showed a reduction of the mean lung dose from 8.27 Gy to 3.18 Gy in favor of proton treatment [31]. The analysis of the dose volume histograms (DVH) revealed that dose reduction was mainly attained by minimizing low dose areas. However, an optimal sparing of the lungs is often accomplished at the expense of a higher heart dose [23]. Several studies observed that radiotherapy potentially impairs functional cardiac parameters such as myocardial perfusion of the inferior left ventricle [32] or cardiac volume with hemodynamic impact [33]. Cardiac toxicity is probably underestimated in esophageal irradiation [34], but the level of clinical relevance of cardiac long-terms effects is unclear due to the limited prognosis of esophageal cancer. For both, pulmonary and cardiac toxicity, CIR has the potential to reduce mean organ doses hence improving the incidence and degree of side effects.

We presented suitable beam configurations for a carbon ion boost to the primary site in the esophagus. Valid and robust CIR for larger volumes like the regional lymphatic pathways treated with elective nodal irradiation (ENI) according to the current recommendations is considerably harder to achieve. However, in case of a further validation and more evidence for the safety of involved-field radiotherapy (IF-RT) in esophageal cancer patients resulting in smaller treatment volumes [35, 36], CIR has the potential to suitably cover tumor boost as well as lymphatic target volumes.

Clinical experience with particle radiotherapy for esophageal cancer is small. At MD Anderson Cancer Center Lin et al. treated 62 patients with proton radiotherapy combined with simultaneous chemotherapy in a definitive or neoadjuvant setting up to a median dose of 50.4 Gy and presented very low toxicity rates [37]. A phase I/II trial from Japan offers the only clinical experience of CIR in esophageal cancer patients available today [12]. In a neoadjuvant setting 31 patients were treated with cumulative doses between 28.8 Gy (RBE) and 36.8 Gy (RBE) in 8 fractions. An encouraging rate of pathological complete remission of 38.7% could be achieved with very low toxicity at the same time. However, PTV margins were defined rather small (GTV + 30 mm for cranial and caudal borders) and dose schemes do not meet current recommendations. Further research is necessary to define effective and safe dosage regimens for CIR. Special attention has to be paid to the esophagus itself as an organ at risk as well as the stomach for distal tumors being prone to complications with increased radiation dose [38].

## Conclusion

Carbon ion radiotherapy for esophageal cancer is feasible with robust dose calculations when variable, intrafractional factors are considered that potentially impair plan quality. Depending on localization and size of the tumor there are several beam configurations that allow an adequate dose coverage and sparing of the organs at risk at the same time. In the future, prospective and randomized trials are necessary to determine the clinical impact of carbon ion radiotherapy on outcome and toxicity.

## Abbreviations

CI: Conformity index
CIR: Carbon ion radiotherapy
CT: Computed tomography-based
DVH: Dose volume histograms
ENI: Elective nodal irradiation
GA: Gantry angle
GTV: Gross tumor volume
HI: Homogenity index
HU: Hounsfield Units
IF-RT: Involved-ield radiotherapy
IMRT: Intensity-modulated radiotherapy
ITV: Internal target volume
LEM: Local effect model
LET: Linear energy transfer (LET)
OAR: Organs at risk
PTV: Planning target volume
RBE: Relative biological effectiveness
SOBP: Spread-out Bragg peak

## References

[1] Sjoquist KM, Burmeister BH, Smithers BM, Zalcberg JR, Simes RJ, Barbour A, Gebski V, Group AG-IT (2011). Survival after neoadjuvant chemotherapy or chemoradiotherapy for resectable oesophageal carcinoma: an updated meta-analysis. Lancet Oncol.

[2] Kranzfelder M, Schuster T, Geinitz H, Friess H, Buchler P (2011). Meta-analysis of neoadjuvant treatment modalities and definitive non-surgical therapy for oesophageal squamous cell cancer. Br J Surg.

[3] Minsky BD, Pajak TF, Ginsberg RJ, Pisansky TM, Martenson J, Komaki R, Okawara G, Rosenthal SA, Kelsen DP (2002). INT 0123 (Radiation Therapy Oncology Group 94–05) phase III trial of combined-modality therapy for esophageal cancer: high-dose versus standard-dose radiation therapy. J Clin Oncol.

[4] Crosby T, Hurt CN, Falk S, Gollins S, Mukherjee S, Staffurth J, Ray R, Bashir N, Bridgewater JA, Geh JI, Cunningham D, Blazeby J, Roy R, Maughan T, Griffiths G (2013). Chemoradiotherapy with or without cetuximab in patients with oesophageal cancer (SCOPE1): a multicentre, phase 2/3 randomised trial. Lancet Oncol.

[5] Carrington R, Spezi E, Gwynne S, Dutton P, Hurt C, Staffurth J, Crosby T (2016). The influence of dose distribution on treatment outcome in the SCOPE 1 oesophageal cancer trial. Radiat Oncol.

[6] Roeder F, Nicolay NH, Nguyen T, Saleh-Ebrahimi L, Askoxylakis V, Bostel T, Zwicker F, Debus J, Timke C, Huber PE (2014). Intensity modulated radiotherapy (IMRT) with concurrent chemotherapy as definitive treatment of locally advanced esophageal cancer. Radiat Oncol.

[7] Kamada T, Tsujii H, Blakely EA, Debus J, De Neve W, Durante M, Jakel O, Mayer R, Orecchia R, Potter R, Vatnitsky S, Chu WT (2015). Carbon ion radiotherapy in Japan: an assessment of 20 years of clinical experience. Lancet Oncol.

[8] Jensen AD, Nikoghosyan AV, Poulakis M, Hoss A, Haberer T, Jakel O, Muenter MW, Schulz-Ertner D, Huber PE, Debus J (2015). Combined intensity-modulated radiotherapy plus raster-scanned carbon ion boost for advanced adenoid cystic carcinoma of the head and neck results in superior locoregional control and overall survival. Cancer.

[9] Kano M, Yamada S, Hoshino I, Murakami K, Akutsu Y, Sakata H, Nishimori T, Usui A, Miyazawa Y, Kamada T, Tsujii H, Matsubara H (2009). Effects of carbon-ion radiotherapy combined with a novel histone deacetylase inhibitor, cyclic hydroxamic-acid-containing peptide 31 in human esophageal squamous cell carcinoma. Anticancer Res.

[10] Takahashi A, Yano T, Matsumoto H, Wang X, Ohnishi K, Tamamoto T, Tsuji K, Yukawa O, Ohnishi T (1998). Effects of accelerated carbon-ions on growth inhibition of transplantable human esophageal cancer in nude mice. Cancer Lett.

[11] Kitabayashi H, Shimada H, Yamada S, Yasuda S, Kamata T, Ando K, Tsujii H, Ochiai T (2006). Synergistic growth suppression induced in esophageal squamous cell carcinoma cells by combined treatment with docetaxel and heavy carbon-ion beam irradiation. Oncol Rep.

[12] Akutsu Y, Yasuda S, Nagata M, Izumi Y, Okazumi S, Shimada H, Nakatani Y, Tsujii H, Kamada T, Yamada S, Matsubara H (2012). A phase I/II clinical trial of preoperative short-course carbon-ion radiotherapy for patients with squamous cell carcinoma of the esophagus. J Surg Oncol.

[13] Kang Y, Zhang X, Chang JY, Wang H, Wei X, Liao Z, Komaki R, Cox JD, Balter PA, Liu H, Zhu XR, Mohan R, Dong L (2007). 4D Proton treatment planning strategy for mobile lung tumors. Int J Radiat Oncol Biol Phys.

[14] Engelsman M, Rietzel E, Kooy HM (2006). Four-dimensional proton treatment planning for lung tumors. Int J Radiat Oncol Biol Phys.

[15] Steidl P. Gating for scanned ion beam therapy. PhD thesis (TU Darmstadt/GSI Helmholtzzentrum für Schwerionenforschung, Germany). 2011.

[16] Wu Q, Mohan R, Morris M, Lauve A, Schmidt-Ulrich R (2003). Simultaneous integrated boost intensity- modulated radiotherapy for locally advanced head-and-neck squamous cell carcinomas. I: dosimetric results. Int J Radiat Oncol Biol Phys.

[17] Kataria T, Sharma K, Subramani V, Karrthick KP, Bisht SS (2012). Homogeneity index: an objective tool for assessment of conformal radiation treatments. J Med Phys.

[18] Dieleman EM, Senan S, Vincent A, Lagerwaard FJ, Slotman BJ, van Sornsen de Koste JR (2007). Four-dimensional computed tomographic analysis of esophageal mobility during normal respiration. Int J Radiat Oncol Biol Phys.

[19] Yaremko BP, Guerrero TM, McAleer MF, Bucci MK, Noyola-Martinez J, Nguyen LT, Balter PA, Guerra R, Komaki R, Liao Z (2008). Determination of respiratory motion for distal esophagus cancer using four-dimensional computed tomography. Int J Radiat Oncol Biol Phys.

[20] Yamashita H, Kida S, Sakumi A, Haga A, Ito S, Onoe T, Okuma K, Ino K, Akahane M, Ohtomo K, Nakagawa K (2011). Four-dimensional measurement of the displacement of internal fiducial markers during 320-multislice computed tomography scanning of thoracic esophageal cancer. Int J Radiat Oncol Biol Phys.

[21] Wang JZ, Li JB, Wang W, Qi HP, Ma ZF, Zhang YJ, Fan TY, Shao Q, Xu M (2013). Detection of interfraction displacement and volume variance during radiotherapy of primary thoracic esophageal cancer based on repeated four-dimensional CT scans. Radiat Oncol.

[22] Zhao KL, Liao Z, Bucci MK, Komaki R, Cox JD, Yu ZH, Zhang L, Mohan R, Dong L (2007). Evaluation of respiratory-induced target motion for esophageal tumors at the gastroesophageal junction. Radiother Oncol.

[23] Zhang X, Zhao KL, Guerrero TM, McGuire SE, Yaremko B, Komaki R, Cox JD, Hui Z, Li Y, Newhauser WD, Mohan R, Liao Z (2008). Four-dimensional computed tomography-based treatment planning for intensity-modulated radiation therapy and proton therapy for distal esophageal cancer. Int J Radiat Oncol Biol Phys.

[24] Mori S, Inaniwa T, Furukawa T, Zenklusen S, Shirai T, Noda K (2013). Effects of a difference in respiratory cycle between treatment planning and irradiation for phase-controlled rescanning and carbon pencil beam scanning. Br J Radiol.

[25] Mori S, Furukawa T, Inaniwa T, Zenklusen S, Nakao M, Shirai T, Noda K (2013). Systematic evaluation of four-dimensional hybrid depth scanning for carbon-ion lung therapy. Med Phys.

[26] Pan X, Zhang X, Li Y, Mohan R, Liao Z (2009). Impact of using different four-dimensional computed tomography data sets to design proton treatment plans for distal esophageal cancer. Int J Radiat Oncol Biol Phys.

[27] Richter D, Graeff C, Jakel O, Combs SE, Durante M, Bert C (2014). Residual motion mitigation in scanned carbon ion beam therapy of liver tumors using enlarged pencil beam overlap. Radiother Oncol.

[28] Han C, Schiffner DC, Schultheiss TE, Chen YJ, Liu A, Wong JY (2012). Residual setup errors and dose variations with less-than-daily image guided patient setup in external beam radiotherapy for esophageal cancer. Radiother Oncol.

[29] Lee HK, Vaporciyan AA, Cox JD, Tucker SL, Putnam JB, Ajani JA, Liao Z, Swisher SG, Roth JA, Smythe WR, Walsh GL, Mohan R, Liu HH, Mooring D, Komaki R (2003). Postoperative pulmonary complications after preoperative chemoradiation for esophageal carcinoma: correlation with pulmonary dose-volume histogram parameters. Int J Radiat Oncol Biol Phys.

[30] Wang SL, Liao Z, Vaporciyan AA, Tucker SL, Liu H, Wei X, Swisher S, Ajani JA, Cox JD, Komaki R (2006). Investigation of clinical and dosimetric factors associated with postoperative pulmonary complications in esophageal cancer patients treated with concurrent chemoradiotherapy followed by surgery. Int J Radiat Oncol Biol Phys.

[31] Welsh J, Gomez D, Palmer MB, Riley BA, Mayankkumar AV, Komaki R, Dong L, Zhu XR, Likhacheva A, Liao Z, Hofstetter WL, Ajani JA, Cox JD (2011). Intensity-modulated proton therapy further reduces normal tissue exposure during definitive therapy for locally advanced distal esophageal tumors: a dosimetric study. Int J Radiat Oncol Biol Phys.

[32] Gayed IW, Liu HH, Yusuf SW, Komaki R, Wei X, Wang X, Chang JY, Swafford J, Broemeling L, Liao Z (2006). The prevalence of myocardial ischemia after concurrent chemoradiation therapy as detected by gated myocardial perfusion imaging in patients with esophageal cancer. J Nucl Med.

[33] Haj Mohammad N, Kamphuis M, Hulshof MC, Lutkenhaus LJ, Gisbertz SS, Bergman JJ, de Bruin-Bon HA, Geijsen ED, Bel A, Boekholdt SM, van Laarhoven HW (2015). Reduction of heart volume during neoadjuvant chemoradiation in patients with resectable esophageal cancer. Radiother Oncol.

[34] Beukema JC, van Luijk P, Widder J, Langendijk JA, Muijs CT (2015). Is cardiac toxicity a relevant issue in the radiation treatment of esophageal cancer?. Radiother Oncol.

[35] Li M, Zhang X, Zhao F, Luo Y, Kong L, Yu J (2016). Involved-field radiotherapy for esophageal squamous cell carcinoma: theory and practice. Radiat Oncol.

[36] Uchinami Y, Myojin M, Takahashi H, Harada K, Shimizu S, Hosokawa M (2016). Prognostic factors in clinical T1N0M0 thoracic esophageal squamous cell carcinoma invading the muscularis mucosa or submucosa. Radiat Oncol.

[37] Lin SH, Komaki R, Liao Z, Wei C, Myles B, Guo X, Palmer M, Mohan R, Swisher SG, Hofstetter WL, Ajani JA, Cox JD (2012). Proton beam therapy and concurrent chemotherapy for esophageal cancer. Int J Radiat Oncol Biol Phys.

[38] Carrington R, Staffurth J, Warren S, Partridge M, Hurt C, Spezi E, Gwynne S, Hawkins MA, Crosby T (2015). The effect of dose escalation on gastric toxicity when treating lower oesophageal tumours: a radiobiological investigation. Radiat Oncol.

